# Cranial meningioma with bone involvement: surgical strategies and clinical considerations

**DOI:** 10.1007/s00701-023-05535-4

**Published:** 2023-03-06

**Authors:** Abigail L. Clynch, Max Norrington, Mohammad A. Mustafa, George E. Richardson, John A. Doherty, Thomas J. Humphries, Conor S. Gillespie, Sumirat M. Keshwara, Catherine J. McMahon, Abdurrahman I. Islim, Michael D. Jenkinson, Christopher P. Millward, Andrew R. Brodbelt

**Affiliations:** 1 School of Medicine, University of Liverpool, Brownlow Hill, Liverpool, L69 7ZX UK; 2 The Walton Centre NHS Foundation Trust, Lower Lane, Fazakerley, L9 7LJ Liverpool UK; 3 Institute of Systems, Molecular and Integrative Biology, University of Liverpool, Brownlow Hill, Liverpool, L69 7ZX UK

**Keywords:** Cranioplasty, Meningioma, Bone involvement, Surgical technique

## Abstract

**Background:**

Intracranial meningioma with bone involvement and primary intraosseous meningioma is uncommon. There is currently no consensus for optimal management. This study aimed to describe the management strategy and outcomes for a 10-year illustrative cohort, and propose an algorithm to aid clinicians in selecting cranioplasty material in such patients.

**Methods:**

A single-centre, retrospective cohort study (January 2010–August 2021). All adult patients requiring cranial reconstruction due to meningioma with bone involvement or primary intraosseous meningioma were included. Baseline patient and meningioma characteristics, surgical strategy, and surgical morbidity were examined. Descriptive statistics were performed using SPSS v24.0. Data visualisation was performed using R v4.1.0.

**Results:**

Thirty-three patients were identified (mean age 56 years; SD 15) There were 19 females. Twenty-nine patients had secondary bone involvement (88%). Four had primary intraosseous meningioma (12%). Nineteen had gross total resection (GTR; 58%). Thirty had primary ‘on-table’ cranioplasty (91%). Cranioplasty materials included pre-fabricated polymethyl methacrylate (pPMMA) (*n* = 12; 36%), titanium mesh (*n* = 10; 30%), hand-moulded polymethyl methacrylate cement (hPMMA) (*n* = 4; 12%), pre-fabricated titanium plate (*n* = 4; 12%), hydroxyapatite (*n* = 2; 6%), and a single case combining titanium mesh with hPMMA cement (*n* = 1; 3%). Five patients required reoperation for a postoperative complication (15%).

**Conclusion:**

Meningioma with bone involvement and primary intraosseous meningioma often requires cranial reconstruction, but this may not be evident prior to surgical resection. Our experience demonstrates that a wide variety of materials have been used successfully, but that pre-fabricated materials may be associated with fewer postoperative complications. Further research within this population is warranted to identify the most appropriate operative strategy.

**Supplementary Information:**

The online version contains supplementary material available at 10.1007/s00701-023-05535-4.

## Introduction

Meningioma bone involvement can occur due to hyperostosis, overt neoplastic infiltration, or primary intraosseous meningioma. Hyperostosis of the cranium is reported in 4.5–17% of cases of intracranial meningioma [32, 38] and is associated with, but not indicative of, tumour cell infiltration [18, 21, 39]. Bone involvement has been observed across all WHO grades of meningioma [13]. For WHO grade 2, bone involvement is associated with an increased risk of recurrence and mortality [16], but little else has been published about the broader prognostic significance. Primary intraosseous meningioma describes an uncommon, distinct subclass of primary extradural meningioma which originates from within the cranial bone [51, 52].

A variety of strategies have been described to surgically manage meningioma with bone involvement. In cases of modest hyperostosis, visible hyperostotic bone can be drilled from the inner table before replacing the refashioned bone flap [28]. Craniectomy in association with meningioma resection may be anticipated if, radiologically, tumour is seen to traverse partly or completely through the associated bone. However, unplanned craniectomy may be necessary in cases where the extent of bony involvement is underestimated preoperatively, and only appreciated on examination of the bone flap, or after drilling the affected area intraoperatively. In such cases, cranial reconstruction is usually required to provide neuroprotection, restore cosmesis, and potentially prevent cognitive decline. Descriptions of clinical outcomes for patients receiving either cranioplasty [5, 12, 44, 48] or replacement of a refashioned hyperostotic bone flap [44] after meningioma resection are limited to case reports and small case series to date.

Following decompressive craniectomy for trauma, cranioplasty has been shown to have a positive impact on social performance, neurocognition, and psychology [1, 14]. Less is known about such benefits in patients requiring cranioplasty for tumour. Cranioplasty is reported to have a relatively high overall complication rate (20–31%), with infection the commonest in non-autologous implants [6, 55]. Additional considerations for tumour patients include the possible need for reoperation or radiotherapy, cosmetic issues caused by the surgery, and the elective nature of treatment allowing implant pre-planning.

Alloplastic cranioplasties are manufactured from a variety of materials including titanium (in mesh, plate, or composite form), ceramics, such as hydroxyapatite (HA), and acrylics such as pre-fabricated polymethyl methacrylate (pPMMA), and hand-moulded polymethyl methacrylate cement (hPMMA) [56]. For cases in which craniectomy is anticipated prior to undertaking surgical resection, pre-fabricated cranioplasty utilising cranial imaging is commonplace. For cases of unplanned craniectomy, a cranioplasty can be fashioned intraoperatively using titanium mesh or hPMMA cement, or performed as a ‘second-stage’ delayed procedure if a pre-fabricated solution is desired. Craniectomy dimensions may be template guided for premade implants where the tumour is in situ, or simply estimated. There are advantages and disadvantages for all materials and methods, and additional considerations are required in cases of reconstruction for tumour. The most suitable technique will depend on tumour location, craniectomy size, patient preference, financial constraints, and the surgeon’s preference and/or familiarity with available materials [56].

The primary aim of this study was to investigate the treatment strategies and outcomes in patients undergoing cranial reconstruction for meningioma with bone involvement. The secondary aim of this study was to propose an algorithm to aid clinicians’ choice of cranioplasty material in these patients.

## Methods

### Study design and inclusion criteria

A single-centre, retrospective cohort study was performed. All adult patients (≥ 16 years of age) who had cranioplasty following surgical resection of intracranial meningioma with bone involvement between January 1st 2010 and August 31st 2021 were included. Cases of primary surgical resection with ‘on-table’ cranioplasty and delayed cranial reconstruction were eligible for inclusion. Patients with meningioma with bone involvement that did not require a cranioplasty were excluded.

### Patient selection

Eligible patients were identified from a pre-existing database of cranial reconstruction cases, previously generated from theatre records and implant registries. The case notes, imaging, operative details, histopathology, and clinical outcomes of all patients were examined. Patients who underwent cranioplasty after meningioma resection for reasons other than bone involvement (e.g. postoperative bone flap infection) were excluded.

### Study variables

Baseline patient characteristics (age, sex, preoperative WHO performance status [PS] and preoperative age-adjusted Charlson co-morbidity index [ACCI]), meningioma characteristics (International Consortium on Meningioma [ICOM] location, diameter, nature of bone involvement, WHO grade, and histopathological subtype), surgical strategy (operative strategy, cranioplasty material, and Simpson grade of resection) [8, 23, 29, 36]. Nature of bone involvement was described as primary intraosseous meningioma or secondary bone involvement. Primary intraosseous meningioma was defined as an absence of an obvious dural origin, where the main tumour mass was within the bone. Secondary bone involvement was defined as the radiological or intraoperative appearance of bone hyperostosis, or histopathological evidence of neoplastic infiltration extending from a primary or dural origin. Extracranial extension was defined as expansion or extrusion through the outer table of the calvarium [12, 42]. Evidence of bone involvement preoperatively was defined as present if reported by a neuro-radiologist in the preoperative scan. Simpson grade was determined from the clinical notes or radiological scans. Use of adjuvant radiotherapy (pre- or post-craniectomy) was recorded. Follow-up time from cranioplasty was recorded.

### Study outcomes

The primary outcome was surgical morbidity associated with the insertion, removal, or replacement of a cranioplasty. Secondary outcomes included surgical morbidity stratified by cranioplasty surgical strategy (intraoperatively designed vs pre-fabricated), and surgical morbidity stratified by timing (primary ‘on table’ vs delayed ‘second stage’). Morbidity was classified using the Therapy-Disability-Neurology (TDN) complication score [53]. The TDN score is a novel scoring system used to classify adverse events in neurosurgery. Events are graded from 1 to 5, with grade 1 representing complications not requiring treatment and grade 5 representing a complication resulting in mortality [54] (Table 1).

**Table 1:** Table summarising the grading of the TDN system [53]

*TDN grade*	*Treatment/intervention*	*Activity of daily living*	*Neurological deficit*
*1*	*Not needed*	*Not affected*	*No new deficits*
*2*	*Pharmacological only*	*Hindering only one*	*A new neurological deficit*
*3*	*Invasive procedure*	*Unable to care for own bodily needs*	*Unable to care for own bodily needs*
*4*	*Intensive care management*	*Bedridden*	*Incontinence*
*5*	*Patient deceased*	*Patient deceased*	*Patient deceased*

### Statistical analysis

Data analysis was performed using SPSS v24.0. Categorical variables are summarised with frequencies and percentages. The distribution of continuous variables was assessed using histograms. Normally distributed variables are expressed as mean (standard deviation (SD)) whereas skewed variables are expressed as median (interquartile range [IQR]). Data visualisation was performed using R v4.1.0.

### Ethical approval

Institutional review board approval was given to conduct this study prior to patient identification. This work falls within the remit of a service evaluation, and therefore individual patient consent was not required.

## Results

### Study population

**Fig. 1:**
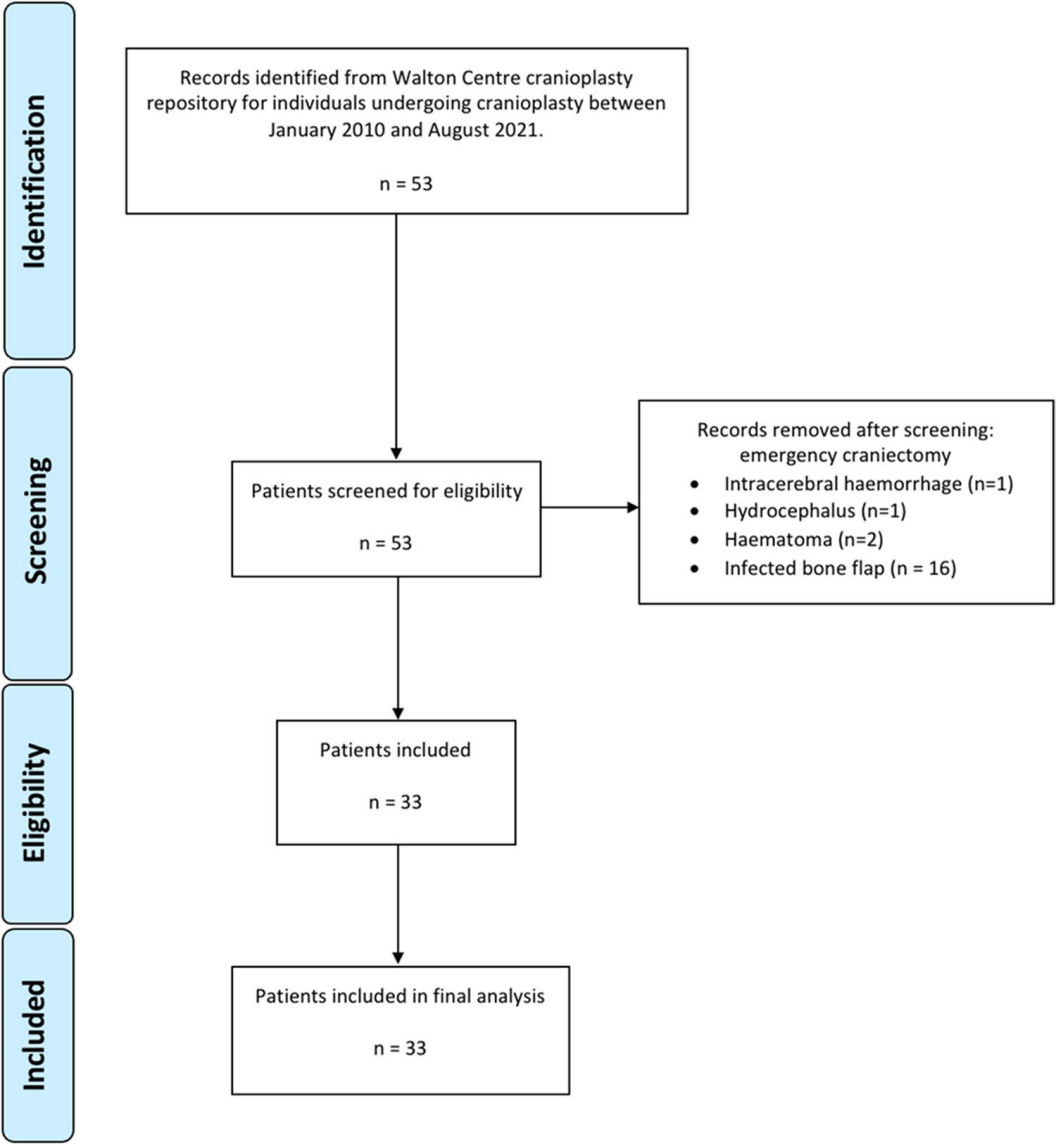
Flow diagram demonstrating the patient selection process for this study—Word document

Thirty-three patients (19 female, 58%) were included in this study. At the time of cranial reconstruction, mean age was 56 (SD = 15). Median follow-up time was 34 months (IQR 9.5–94). Figure 1 illustrates the patient selection process and Supplementary Table 1 summarises the study cohort.

### Meningioma characteristics

The most common ICOM location was convexity (*n* = 16, 49%). Mean tumour diameter was 49.3 mm (SD = 18). Seventeen meningioma were WHO grade 1 (52%). Sixteen meningioma were WHO grade 2 (48%). One patient had multiple meningioma.

Twenty-nine patients had meningioma with bone involvement (88%) whilst four had primary intraosseous meningioma (12%). On imaging, twenty-one patients had extracranial extension (64%).

### Surgical strategy

Surgical strategies are summarised in Supplementary Table 1 and described at an individual patient level in Supplementary Table 2.

Eighteen patients had insertion of a pre-fabricated cranioplasty (55%). Pre-fabricated materials included pPMMA (*n* = 12; 36%), titanium plate (*n* = 4; 12%), and HA (*n* = 2; 6%). Fifteen patients had an intraoperatively designed cranioplasty (46%), which included titanium mesh (*n* = 10; 30%), hPMMA (*n* = 4; 112%), and a single case of combined titanium mesh and hPMMA (3%).

Thirty patients had primary ‘on-table’ cranioplasty following tumour resection (91%). Three patients had a delayed cranioplasty following tumour resection as a ‘second-stage’ procedure (9%). Of the patients receiving primary ‘on-table’ cranioplasty, twenty-three patients were identified as having bone involvement on radiological scans prior to the operation, eleven of whom received a pre-fabricated cranioplasty (48%). Of the patients receiving delayed ‘second-stage’ cranioplasty, all patients received a pre-fabricated cranioplasty. Reasons for delayed cranioplasty were not recorded in one patient (33%). Two patients underwent delayed cranioplasty due to intraoperative brain swelling (67%).

### Surgical morbidity and mortality

Nineteen patients suffered a postoperative complication, of whom eight patients had complications classified as TDN grade 3 to 5. Four patients suffered more than one postoperative complication. Based on the highest TDN grade per patient, two patients had a complication TDN grade 5 (6%), six patients had a complication TDN grade 3 (18%), four patients had a complication TDN grade 2 (12%), and five patients had a complication TDN grade 1 (15%). The most common surgical complications were pseudomeningocele (*n* = 4; 12%), sensory disturbance (*n* = 4; 12%), and seizures (*n* = 4; 12%). Other complications patients experienced postoperatively were DVT/PE (*n* = 3; 9%), CSF leak (*n* = 2; 6%), haematoma (*n* = 2; 6%), pneumonia (*n* = 2; 6%), wound infection (*n* = 1; 3%), cosmetic complaint (*n* = 1; 3%), dysphasia (*n* = 1; 3%), wound erosion (*n* = 1; 3%), and incontinence (*n* = 1: 3%).

Two patients died < 30 days postoperatively. Both patients died from postoperative pneumonia (Study Number 3 and 20). Both patients had poor preoperative PS and above average ACCI compared to the remaining cohort.

### Reoperation

Five patients underwent reoperation due to postoperative complications (15%). Causes of reoperation were repair of pseudomeningocele (*n* = 2; 29%), wound erosion (*n* = 1; 14%), wound infection (*n* = 1; 14%), and haematoma (*n* = 1; 14%). No patients had further complications following reoperation. Two patients underwent reoperation for tumour recurrence (6%).

### Morbidity outcomes stratified by material type (intraoperatively designed vs pre-fabricated)

**Fig. 2:**
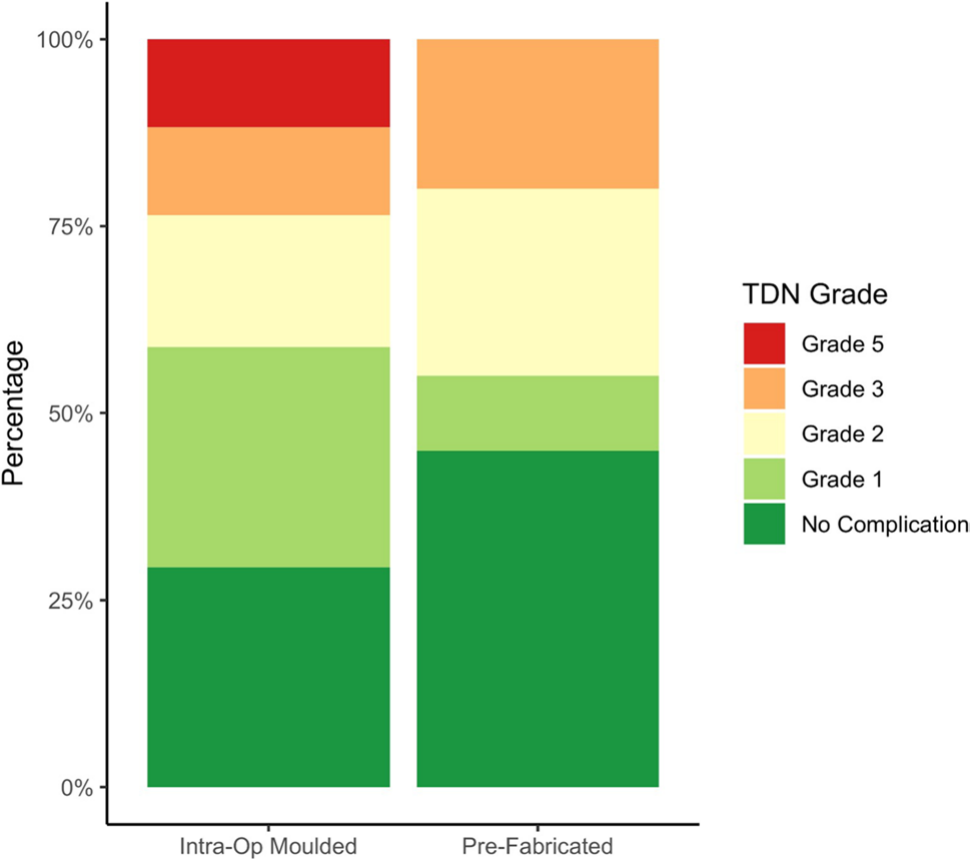
Stacked bar chart showing TDN complication rates in this cohort by material (intraoperatively designed vs pre-fabricated)—produced using R

Of patients receiving a pre-fabricated cranioplasty, nine patients had a postoperative complication. Five patients had more than one postoperative complication. Based on the highest TDN grade per patient, four patients had a complication classified as TDN grade 3 (22%), three had a complication classified as TDN grade 2 (17%), and two had a complication classified as TDN grade 1 (11%). Of patients receiving an intraoperatively designed cranioplasty, ten patients had a postoperative complication. Two patients had more than one postoperative complication. Based on highest TDN grade per patient, two patients had a complication classified as TDN grade 5 (13%), two patients had a complication classified as TDN grade 3 (13%), two patients had a complication classified as TDN grade 2 (13%), and four patients had a complication classified as TDN grade 1 (27%). Figure 2 illustrates surgical morbidity stratified by material type using TDN scores.

### Morbidity outcomes stratified by timing of cranioplasty (primary ‘on-table’ cranioplasty vs delayed ‘second-stage’ cranioplasty)

One patient with a delayed cranioplasty had a postoperative complication compared to seventeen patients who underwent on table cranioplasty (33% vs 57%). Patients who underwent on table cranioplasty had complications with TDN grade 5 (*n* = 2; 7%), grade 3 (*n* = 6; 20%), grade 2 (*n* = 7; 23%), and grade 1 (*n* = 9; 27%). The complication following delayed cranioplasty was TDN grade 2 (n = 1; 33%).

## Discussion

This study describes a cohort of thirty-three patients over a 10-year period that required cranial reconstruction following meningioma resection. Most cases were of intracranial meningioma with secondary bone involvement who underwent primary ‘on table’ cranioplasty at the time of meningioma resection. Similar numbers of patients received either a pre-fabricated or intraoperatively fashioned cranioplasty. Five patients underwent reoperation for a postoperative complication. When compared to patients receiving intraoperatively moulded cranioplasty, those with pre-fabricated materials experienced fewer morbidity events. Due to limited cases of delayed ‘second-stage’ cranioplasty, we were unable to evaluate morbidity outcomes stratified by timing of cranioplasty.

Management of meningioma with bone involvement is still widely debated. Extent of resection of meningioma is a prognostic factor for recurrence and survival [3, 47], and in those cases with overt bone infiltration, craniectomy is often required. When the resulting cranial defect is large, cranial reconstruction is necessary to provide neuroprotection and better cosmesis. For patients with hyperostosis of the bone without obvious radiological evidence of bone invasion, the need for craniectomy and cranioplasty reconstruction is less clear, and replacement of a refashioned bone flap is often undertaken, rather than replacement with an alloplastic cranioplasty. Clinicians must balance the risk of recurrence due to subtotal resection of tumour involved bone with the complications associated with alloplastic cranioplasty, either as a single- or two-stage procedure. In making such decisions, clinicians must consider several factors including operative planning scans, cranioplasty technique, and intraoperative considerations. This study focussed solely on alloplastic reconstruction.

### Operative planning scan

Preoperative magnetic resonance imaging (MRI) and computed tomography (CT) scans are performed to assess the extent of bony involvement. These scans are used to inform operative planning and to produce custom-made pre-fabricated cranioplasties. Such imaging techniques provide limited diagnostic value and may not show the true extent of bony involvement [2, 4, 19, 51]. This presents an issue particularly when an expensive, pre-fabricated implant has been created around the diameters provided from MRI and CT scans. Recently, developments in imaging techniques have suggested that positron emission tomography (PET) scans, notably ^68^ Ga-DOTATATE PET, are particularly useful in operative planning [9, 17, 36, 46]. A study examining the use of ^68^ Ga-DOTATATE PET and contrast-enhanced MRI (CE MRI) in identifying intraosseous involvement of meningioma found that GA-DOTATE PET was significantly better at predicting preoperative bone involvement compared to CE MRI [26]. However, this imaging modality is not standard practice within neurosurgical centres. In light of this, it raises the clinical question – how confident can we be when imaging shows simple hyperostosis and how accurate are current imaging techniques in identifying bony involvement? This uncertainty must be considered by clinicians prior to any decision-making concerning cranioplasty technique, particularly in imaging where bony involvement is not well demarcated.

### Cranioplasty technique

Alloplastic materials can be classed as those that are pre-fabricated (e.g. pPMMA, HA, and titanium plates) and those that are intraoperatively fashioned (e.g. hPMMA and titanium mesh). When bone involvement is anticipated preoperatively, surgeons may elect to choose a pre-fabricated cranioplasty solution, or proceed with an intraoperatively designed cranioplasty. Pre-planning allows clinicians the opportunity to take advantage of the benefits associated with the use of pre-fabricated materials including a good functional fit, improved cosmesis, and reduced infection and subsequent explanation rate [11, 35, 42]. Finally, pre-fabricated materials can address issues relating to exothermic reactions and tissue necrosis that have been reported with intraoperatively designed materials [56]. Disadvantages of pre-fabricated material include an increased cost, and the time lag for production (unless cranioplasty is performed as a second-stage procedure) leaving patients symptomatic for longer periods and risking further growth of the meningioma. Production times have dramatically reduced, and most suppliers now have a lag time of only 2 weeks.

Intraoperatively fashioned cranioplasty offers the benefits of a quicker solution at a lower cost [42]. Similarly, pre-fabricated material, HA carries a risk of implant fracture and a higher rate of postoperative epidural haematoma compared to titanium cranioplasty [29, 48]. Furthermore, HA has been demonstrated in orthopaedic surgery to successfully osteointegrate with adjacent bone, in a phenomenon described as osteotropism [22]. Whilst in head injury osteointegration can be seen as an advantage, in meningioma surgery, it is important to consider the possible need for reoperation, where removal of an osteointegrated cranioplasty may be more difficult. hPMMA implants cannot be infiltrated by new bone tissue [56].

Titanium cranioplasty has one of the highest infection and cranioplasty revision rates, as per the recent network meta-analysis by Henry et al., although this was not evident in the series presented here [23]. Titanium is an on-lay material and as a result provides a high degree of strength when used for cranioplasty. In circumstances where swelling is anticipated, the added space created using an on-lay material may be preferable to an inlay cranioplasty. Titanium mesh allows for an intraoperative extension to the craniectomy for bone infiltration beyond the initial operative plan, which is difficult with other pre-fabricated inlay materials. Silver-coated titanium has been developed and reported to have a lower infection rate than titanium alone. No implant is watertight, titanium less so than others, and a watertight dural closure should be considered in all patients if possible. Titanium produces more local artefact with CT and MRI imaging compared to all other cranioplasty materials, making the detection of small recurrences more difficult on follow-up imaging.

Surgical morbidity is an important preoperative consideration for clinicians. The three most common complications in our cohort were pseudomeningocele, seizures, and sensory disturbance. The most frequently reported complications of cranioplasty in the literature are pseudomeningocoele, seizure, and CNS infection [24]. Infection usually results in explantation of the cranioplasty. The reported incidence of cranioplasty infection ranges from 7.1 to 26% [7, 55]. In this cohort, only one patient (who also had a postoperative external ventricular drain) developed a superficial wound infection. This patient underwent cranioplasty explantation. The single incidence precludes comment on the relevance of the cranioplasty material and technique for this infection. Pseudomeningocele was the most common cause of reoperation due to a postoperative complication in this cohort. There are no standardised guidelines for the management of pseudomeningoceles after meningioma resection—some may resolve spontaneously whilst others may require operative intervention [40, 53]. Risk factors for persistent pseudomeningoceles include infection, radiation, malnutrition, and increased CSF pressure [10]. Clinicians should consider this risk particularly in immunosuppressed patients, or those with tumour recurrence following radiation therapy. When morbidity was stratified by pre-fabricated and intraoperative cranioplasty, our data demonstrated a trend towards less morbidity, and morbidity of lower severity, when pre-fabricated materials were used. This is in keeping with current literature [42].

### Proposed algorithm

**Fig. 3:**
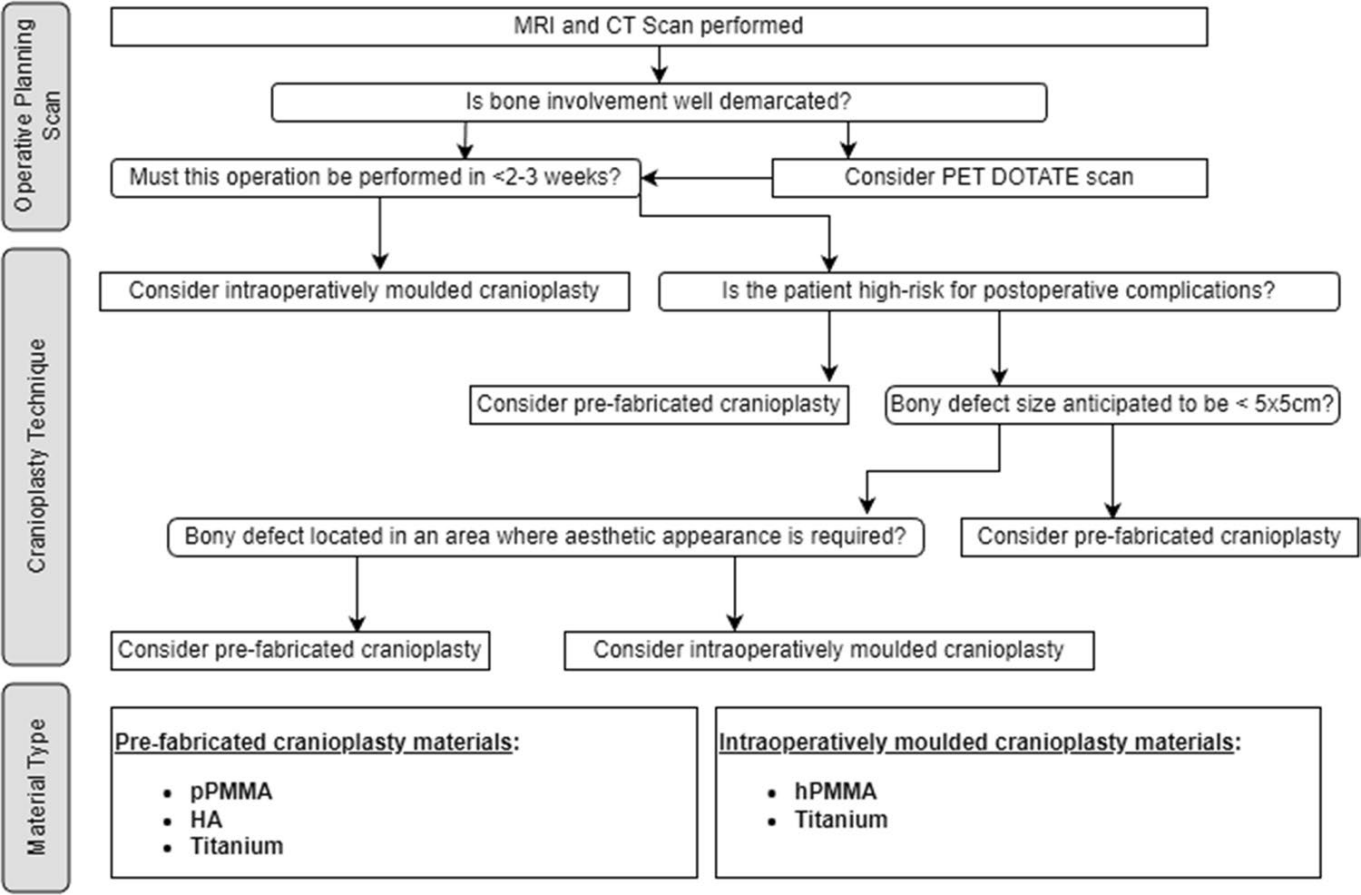
Proposed algorithm for selecting cranioplasty material in meningioma patients with bone involvement – produced using draw.io

Following examination of the clinical literature and the results of our study, we formulated a proposed algorithm for selecting cranioplasty material. This is depicted in Fig. 3. When examining operative planning scans, clinicians must first consider if the image confidently provides them with a well-demarcated area of bone involvement. If this is the case, then intraoperative discarding of a pre-fabricated cranioplasty is unlikely and as such a pre-fabricated cranioplasty may be selected. However, if a patient requires urgent meningioma resection, then an intraoperatively moulded cranioplasty is more appropriate. In patients who do not require urgent surgery, but whose operation is for removal of a tumour recurrence, clinicians may wish to avoid titanium cranioplasty were possible. Intraoperatively, if patient’s bone involvement is not as anticipated, clinicians must consider if their patient can undergo a second-stage delayed cranioplasty. If they cannot, then clinicians may choose to insert an intraoperatively moulded cranioplasty.

### Limitations of this study

There are a number of limitations to this study. The cohort size is relatively small, limiting statistical exploration. The low number of infections and explantation precluded comments on cranioplasty timing and material. Limited data were available on the indications for the four patients who had delayed cranial reconstruction.

## Conclusion

Meningioma with bone involvement and primary intraosseous meningioma often require cranial reconstruction. A wide variety of materials have been used successfully. Pre-fabricated materials are associated with fewer postoperative complications, but require pre-planning and have a lead time for cranioplasty production. Although titanium has a higher reported infection rate in the literature, there are some advantages in patients with meningiomas with bone involvement. A simple algorithm may help surgeons decide the appropriate material and timing of surgery.

## Supplementary Information

Below is the link to the electronic supplementary material.

Supplementary file1 (DOCX 60 KB)

## Data Availability

Anonymised data from our patient cohort can be available, where appropriate, on request from the joint first authors.

## Abbreviations

GTR: Gross total resection
hPMMA: Hand-moulded polymethyl methacrylate
HA: Hydroxyapatite
pPMMA: Pre-fabricated polymethyl methacrylate
WHO: World Health Organization
UK: United Kingdom
PS: Performance status
ACCI: Age-adjusted Charlson co-morbidity index
ICOM: International Consortium on Meningioma
TDN: Therapy-disability-neurology
SD: Standard deviation
IQR: Interquartile range
IRB: Institutional Review Boards
CSF: Cerebrospinal fluid
