# Correction to: Cranial meningioma with bone involvement: surgical strategies and clinical considerations

**DOI:** 10.1007/s00701-023-05710-7

**Published:** 2023-07-21

**Authors:** Abigail L. Clynch, Max Norrington, Mohammad A. Mustafa, George E. Richardson, John A. Doherty, Thomas J. Humphries, Conor S. Gillespie, Sumirat M. Keshwara, Catherine J. McMahon, Abdurrahman I. Islim, Michael D. Jenkinson, Christopher P. Millward, Andrew R. Brodbelt

**Affiliations:** 1 School of Medicine, University of Liverpool, Brownlow Hill, Liverpool, L69 7ZX UK; 2 The Walton Centre NHS Foundation Trust, Lower Lane, Fazakerley, Liverpool, L9 7LJ UK; 3 Institute of Systems, Molecular and Integrative Biology, University of Liverpool, Brownlow Hill, Liverpool, L69 7ZX UK

**Correction to: Acta Neurochirurgica (2023) 165:1355–1363**

**https://doi.org/10.1007/s00701-023-05535-4**

Reference 53 is incorrect, the correct reference should be:

Terrapon APR, Zattra CM, Voglis S, Velz J, Vasella F, Akeret K, Held U, Schiavolin S, Bozinov O, Ferroli P, Broggi M, Sarnthein J, Regli L, Neidert MC. Adverse Events in Neurosurgery: The Novel Therapy-Disability-Neurology Grade. Neurosurgery. 2021 Jul 15;89(2):236–245. doi:https://doi.org/10.1093/neuros/nyab121. PMID: 33887774.

The original article has been corrected.

