# Supplementary material for: Cranial meningioma with bone involvement: surgical strategies and clinical considerations
Source: Acta Neurochir (Wien). 2023 Mar 6;165(5):1355–63. doi: 10.1007/s00701-023-05535-4 (PMC10140130; doi:10.1007/s00701-023-05535-4)
Supplement: Supplementary file 1 — Supplementary file1 (DOCX 60 KB) [file 701_2023_5535_MOESM1_ESM.docx]

| Variable | Sub-variable | Total number (%)/[IQR]/{SD} |
| --- | --- | --- |
| Baseline Patient Characteristics | | |
| Number of patients | | 33 |
| Mean age at craniectomy (years) | | 56 {15; 28-86} |
| Sex | Male | 14 (42) |
|  | Female | 19 (58) |
| Median pre-cranioplasty WHO performance status | | 1 [0-1] |
| Median pre-cranioplasty ACCI | | 1.0 [1-3] |
| Meningioma Characteristics | | |
| ICOM location | Convexity | 16 (49) |
|  | Parasagittal | 7 (21) |
|  | Parafalcine | 4 (12) |
|  | Anterior midline | 2 (6.1) |
|  | Sphenoid wing | 3 (9.1) |
|  | Supratentorial | 1 (3.0) |
| Mean diameter (mm) | | 49.26 {18} |
| Nature of bone involvement | Primary intraosseous meningioma | 4 (12) |
|  | Secondary bone involvement | 8 (24) |
|  | Secondary bone involvement and extracranial extension | 21 (64) |
| WHO Grade | 1 | 17 (52) |
|  | 2 | 16 (49) |
| Histological subtype | Meningiothelial | 10 (30) |
|  | Transitional | 5 (15) |
|  | Microcystic | 1 (3.0) |
|  | Psammomatous | 1 (3.0) |
|  | Atypical | 13 (39) |
|  | Unreported | 3 (9.1) |
| Surgical strategy | | |
| Operative strategy | Primary surgical resection and on table cranioplasty | 25 (76) |
|  | Recurrence surgical resection and on table cranioplasty | 5 (18) |
|  | Delayed cranioplasty | 3 (6.1) |
| Cranioplasty material | Titanium Mesh | 10 (30) |
|  | Titanium Plate | 4 (12) |
|  | Pre-fabricated acrylic | 12 (36) |
|  | Hand-moulded PMMA | 4 (12) |
|  | Hydroxyapatite | 2 (6.1) |
|  | Combination (titanium mesh + PMMA) | 1 (3.0) |
| Simpson grade of resection | I | 8 (24) |
|  | II | 6 (18) |
|  | III | 5 (15) |
|  | IV | 12 (36) |
|  | Not available | 2 (6.1) |
| Adjuvant radiotherapy | Pre-cranioplasty | 2 (20) |
|  | Post-cranioplasty | 8 (80) |
| Surgical morbidity | | |
| Complications | | 26 |
| Specific complications | Cosmetic complaint | 1 (3.8) |
|  | CSF leak | 2 (7.7) |
|  | Haematoma | 2 (7.7) |
|  | Pseudomeningiocele | 4 (15) |
|  | Wound infection | 1 (3.8) |
|  | Pneumonia | 1 (3.8) |
|  | DVT/PE | 4 (15) |
|  | Wound erosion | 1 (3.8) |
|  | Sensory disturbance | 4 (15) |
|  | Seizures | 4 (15) |
|  | Dysphasia | 1 (3.8) |
|  | Incontinence | 1 (3.8) |
| Median 6-month postoperative performance status | | 1 [0-2] |
| Reoperation | Reoperation for tumour recurrence | 2 (6.1) |
|  | Reoperation for other reason | 5 (15) |
| Median follow-up time post-cranioplasty (months) | | 34 [9.5-94.0] |

**^Table 1. Summary table of patient baseline characteristics, meningioma characteristics, intervention details and postoperative outcomes^**

| Study Number | Baseline Patient characteristics | | | Meningioma Characteristics | | | | Surgical strategy | | | | Surgical morbidity | | | | |
| --- | --- | --- | --- | --- | --- | --- | --- | --- | --- | --- | --- | --- | --- | --- | --- | --- |
|  | Age; sex | **Pre-op PS** | **Pre-op CCI** | **ICOM location** | **Diameter (mm)** | **Nature of Bone involvement**  **(evidence on preoperative scan)** | **WHO Grade and subtype** | **Operative Strategy** | **Cranioplasty material** | **Simpson Grade of resection** | **Adjuvant radiotherapy** | **Complications** | **6-month post-op PS** | **All-cause reason for reoperation** | **Surgical complications post-reoperation** | **Follow-up from cranioplasty (months)** |
| 1 | 55; M | 1 | 1 | Sphenoid wing | 32.7 | Secondary bone involvement + extracranial extension  (no) | Grade 1 - meningiothelial | Primary surgical resection and on table cranioplasty | PMMA;  Hand-moulded | 1 | No | Cosmetic complaint and seizures | 2 | No |  | 130 |
| 2 | 83; F | 1 | 4 | Parasagittal | 48.0 | Secondary bone involvement + extracranial extension  (no) | Grade 1 - meningiothelial | Primary surgical resection and on table cranioplasty | Titanium; mesh | 3 | No | No | 1 | No |  | 32 |
| 3 | 86; M | 2 | 4 | Convexity | 78.3 | Secondary bone involvement + extracranial extension  (no) | Grade 2 – atypical | Primary surgical resection and on table cranioplasty | Titanium; mesh | 1 | No | Pneumonia | 5 | No |  | 0  (deceased) |
| 4 | 70; M | 1 | 3 | Convexity | 83.0 | Secondary bone involvement + extracranial extension  (yes) | Grade 2- atypical | Delayed cranial reconstruction | Titanium;  plate | 4 | Yes (pre-cranioplasty) | DVT | 0 | No |  | 103  (deceased) |
| 5 | 63; M | 1 | 2 | Convexity | 44.0 | Secondary bone involvement + extracranial extension  (yes) | Grade 2- meningiothelial | Primary surgical resection and on table cranioplasty | Titanium;  Mesh | 4 | Yes | CSF leak and wound erosion | 1 | Revision of Cranioplasty due to wound erosion | No | 121 |
| 6 | 65; M | 0 | 2 | Parafalcine | 37.4 | Primary interosseous meningioma  (yes) | Grade 2- atypical | Recurrent surgical resection and on table cranioplasty | Acrylic;  Pre-fabricated | 4 | No | Seizures | 0 | No |  | 69 |
| 7 | 37; F | 0 | 0 | Convexity | 35.0 | Primary interosseous meningioma  (yes) | Grade 1- microcystic | Primary surgical resection and on table cranioplasty | PMMA;  Hand-moulded | 2 | No | No | 0 | No |  | 110 |
| 8 | 60; F | 1 | 2 | Convexity | 61.5 | Secondary bone involvement + extracranial extension  (yes) | Grade 1- meningiothelial | Primary surgical resection and on table cranioplasty | Acrylic;  Pre-fabricated | 1 | No | Hemiplegia and incontinence | 2 | No |  | 100 |
| 9 | 45; F | 1 | 0 | Sphenoid wing | 61.6 | Secondary bone involvement + extracranial extension  (yes) | Grade 1- meningiothelial | Primary surgical resection and on table cranioplasty | Titanium;  Mesh | 2 | No | No | 0 | Removal of cranioplasty due to tumour recurrence, new titanium mesh inserted | No | 103 |
| 10 | 50; M | 1 | 1 | Convexity | 52.8 | Secondary bone involvement + extracranial extension  (yes) | Grade 2- atypical | Primary surgical resection and on table cranioplasty | Titanium;  Mesh | 4 | Yes | CSF leak, weakness and sensory disturbance | 2 | No |  | 19  (deceased) |
| 11 | 41; F | 0 | 0 | Parasagittal | 48.0 | Secondary bone involvement + extracranial extension  (yes) | Grade 1- not reported | Primary surgical resection and on table cranioplasty | Acrylic;  Pre-1fabricated | 3 | No | No | 0 | No |  | 97 |
| 12 | 52; M | 3 | 1 | Convexity | 35.2 | Secondary bone involvement + extracranial extension  (yes) | Grade 1 - meningiothelial | Recurrent surgical resection and delayed cranial reconstruction | Titanium;  Plate | 4 | Yes (pre-cranioplasty) | No | 3 | No |  | 97 |
| 13 | 41; F | 0 | 1 | Convexity | 82.0 | Primary interosseous meningioma  (yes) | Grade 1- meningiothelial | Primary surgical resection and on table cranioplasty | Acrylic;  Pre-fabricated | 1 | No | Pseudomeningocele and seizures | 1 | Repair of pseudomeningiocele | No | 95 |
| 14 | 75; F | 1 | 3 | Convexity | 63.3 | Secondary bone involvement + extracranial extension  (yes) | Grade 2- atypical | Primary surgical resection and on table cranioplasty | PMMA;  Hand-moulded | 2 | No | No | 0 | No |  | 89 |
| 15 | 74; F | 2 | 3 | Parafalcine | 76.0 | Secondary bone involvement + extracranial extension  (yes) | Grade 2- psammomatous | Primary surgical resection and on table cranioplasty | Titanium;  Mesh | 4 | Yes | PE | 3 | No |  | 9 |
| 16 | 53; M | 0 | 1 | Parasagittal | 45.0 | Secondary bone involvement + extracranial extension  (no) | Grade 2- atypical | Primary surgical resection and on table cranioplasty | PMMA;  Hand-moulded | 4 | Yes | Wound infection (K.pneumoniae), pseudomeningocele | 0 | No |  | 33 |
| 17 | 68; M | 2 | 2 | Parafalcine | 69.7 | Secondary bone involvement + extracranial extension  (yes) | Grade 2- atypical | Primary surgical resection and on table cranioplasty | Acrylic;  Pre-fabricated | 4 | Yes | Pseudomeningiocele, hydrocephalus, wound infection, DVT/PE | 4 | Repair of pseudomeningiocele using pseudomeningocele peritoneal shunt, followed by Lumbar drain VP shunt;  Epidural periprosthetic abscess leading to subsequent wash out and cranioplasty explantation | No | 10  (deceased) |
| 18 | 55;F | 2 | 1 | Parasagittal | 57.0 | Secondary bone involvement + extracranial extension  (no) | Grade 2- meningiothelial | Primary surgical resection and on table cranioplasty | Titanium;  Mesh | 4 | No | Haematoma | 5 | Removal of cranioplasty and wash out | No | 0  (deceased) |
| 19 | 57; M | 0 | 1 | Parasagittal | 34.0 | Secondary bone involvement + extracranial extension  (yes) | Grade 1- not reported | Primary surgical resection and on table cranioplasty | Acrylic;  Pre-fabricated | 2 | No | Seizures | 0 | No |  | 63 |
| 20 | 73; M | 2 | 5 | Parafalcine | 47.0 | Secondary bone involvement + extracranial extension  (yes) | Grade 2- atypical | Primary surgical resection and on table cranioplasty | Titanium;  mesh | N/A | No | Pneumonia | 5 | No |  | 1  (deceased) |
| 21 | 43; F | 0 | 0 | Convexity | 30.0 | Secondary bone involvement + extracranial extension  (no) | Grade 1- meningiothelial | Primary surgical resection and on table cranioplasty | Titanium;  Mesh | 2 | Yes | No | 0 | No |  | 49 |
| 22 | 37; F | 0 | 0 | Convexity | 33.9 | Secondary bone involvement  (yes) | Grade 2- atypical | Recurrent surgical resection and on table cranioplasty | acrylic;  Pre-fabricated | N/A | Yes | No | 0 | No |  | 75 |
| 23 | 37; F | 1 | 0 | Parasagittal | 30.0 | Secondary bone involvement + extracranial extension  (yes) | Grade 1- not reported | Primary surgical resection and on table cranioplasty | acrylic;  Pre-fabricated | 4 | No | No | 2 | No |  | 48 |
| 24 | 59; F | 0 | 1 | Anterior midline | 56.0 | Secondary bone involvement + extracranial extension  (yes) | Grade 1- transitional | Primary surgical resection and on table cranioplasty | Titanium;  Mesh  and PMMA cement on top | 3 | No | No | 0 | No |  | 37 |
| 25 | 51; M | 0 | 1 | Convexity | 55.7 | Secondary bone involvement  (yes) | Grade 2- atypical | Primary surgical resection and on table cranioplasty | Acrylic;  pre-fabricated | 1 | No | No | 0 | No |  | 34 |
| 26 | 51; F | 1 | 1 | Anterior midline | 71.0 | Secondary bone involvement  (no) | Grade 1- transitional | Recurrent surgical resection and on-table cranioplasty | Hydroxyapatite | 3 | No | No | 0 | No |  | 46 |
| 27 | 28; M | 0 | 0 | Convexity | 36.0 | Secondary bone involvement  (yes) | Grade 1- transitional | Primary surgical resection and on table cranioplasty | Acrylic;  pre-fabricated | 1 | No | No | 0 | No |  | 26 |
| 28 | 46; F | 0 | 1 | Convexity | 39.0 | Primary interosseous meningioma  (yes) | Grade 1- meningiothelial | Recurrent surgical resection and on table cranioplasty | PMMA;  Hand-moulded | 1 | No | pseudomeningocele | 1 | Surgical repair of pseudomengingocele, removal of cranioplasty and insertion of hydroxyapatite cranioplasty | Swelling and cosmetic complaint | 22 |
| 29 | 69; F | 0 | 2 | Convexity | 56.3 | Secondary bone involvement  (yes) | Grade 2- atypical | Primary surgical resection and on table cranioplasty | PMMA;  Hand-moulded | 2 | No | DVT | 0 | No |  | 7 |
| 30 | 73;  M | 0 | 4 | Parasagittal | 58.9 | Secondary bone involvement + extracranial extension  (yes) | Grade 2- atypical | Primary surgical resection and on-table cranioplasty | Titanium; mesh | 4 | Yes | Weakness and dysphasia | 2 | Removal of cranioplasty due to meningioma regrowth, same cranioplasty reinserted | No | 14 |
| 31 | 51;  F | 0 | 4 | Supratentorial | 23.4 | Secondary bone involvement  (yes) | Grade 1- transitional | Primary surgical resection and on table cranioplasty | Titanium;  Mesh | 4 | No | Hemiparesis and weakness | 1 | No |  | 8 |
| 32 | 57;  F | 0 | 3 | Convexity | 19.0 | Secondary bone involvement  (yes) | Grade 2- atypical | Recurrent surgical resection and on table cranioplasty | Acrylic;  Pre-fabricated | 3 | No | Haematoma | 1 | No |  | 8 |
| 33 | 54;  F | 1 | 1 | Sphenoid wing | 25.0 | Secondary bone involvement  (yes) | Grade 1- transitional | Delayed cranial reconstruction | Hydroxyapatite | 1 | No | No | 1 | No |  | 0 |

**^Table 2:^** **^Demographic characteristics, Meningioma characteristics, intervention details and postoperative outcomes of patients included in this cohort study^**

| Material | Time of design | Method of design | Features | References |
| --- | --- | --- | --- | --- |
| Polymers (PMMA, PEEK) | Pre-made | CAD of cranioplasty and craniotomy template | 1) High chance of conformation and can be drilled to fit if necessary  2) High mechanical resistance  3) Radiolucent and radiotherapy compatible  4) Expensive  5) Good cosmetic result  6) Difficult if intraoperative bone involvement is greater than anticipated.  7) Lag time for production | [5, 11] |
|  | Off-shelf | Cut rim of bone around craniotomy, insert polymer, replace craniotomy flap | 1) Good for small areas of bone  2) Radiolucent and radiotherapy compatible  3) Cheaper  4) Risk of thermal injury  5) Report of tumour cell infiltration (acrylic)  6) Cosmetic result may not be as good as pre-produced models | [22, 26] |
| Titanium | Pre-op | CAD of cranioplasty and craniotomy template  or  CAD of cranioplasty and stereo-lithographic model of skull for planning | 1) Minimal radio-artifact  2) Majority are radiotherapy compatible  3) Expensive  4) Not watertight  5) Allow intraoperative unanticipated extension of craniectomy  6) Strong  7) Higher infection rate reported.  8) Can be silver coated to reduce infection  9) Good cosmetic result | [8, 25, 28] |
|  | Off-shelf | Mesh shaped and cut to skull overlying craniotomy site | 1) Cheaper  2) No planning required  3) Useful in emergency  4) Radiotherapy compatible  5) Not as good cosmetic result with larger defects | [21] |
| Hydroxyapatite | Pre-op | CAD of cranioplasty and craniotomy template | 1) Osteointegration – potential difficulties in explantation  2) Avid if large defect and significant falls risk  3) technically more demanding to insert with little intraoperative flexibility  4) Good cosmetic result | [14] |
| Composite | Off-shelf | Mesh shaped and cut to skull overlying craniotomy site. Cut rim of bone around mesh-covered craniotomy. Polymer inserted beneath mesh and reinsert | 1) Avoids risk of thermal injury  2) More complex procedure  3) Not always provide a good cosmetic result | [4] |

**^Table 3. Cranioplasty methods and materials for meningioma surgery^**
